# Genome sequences of bacteriophage Shambre1 and Renna12, isolated from *Arthrobacter globiformis*

**DOI:** 10.1128/mra.00858-23

**Published:** 2023-12-13

**Authors:** Madeline Dojs, Christine Fleischacker, Skylar Ackerman, Blaise Boyle, Shambre Feiring, Thomas Fleischacker, Jaycee Frank, Sarah Jackson, Ashlin Schaefbauer, Corinna Vigness, Rylie Webb

**Affiliations:** 1 Department of Biology, University of Mary, Bismarck, North Dakota, USA; Loyola University Chicago, Chicago, Illinois, USA

**Keywords:** phage, bacteriophage, bacteriophage genetics

## Abstract

Bacteriophages Shambre1 and Renna12 were isolated from soil in Bismarck, ND, using *Arthrobacter globiformis*. Genomic characterization and analyses allowed Renna12 to be assigned to phage cluster AS3, while Shambre1, which is not closely related to any phage, is a singleton.

## ANNOUNCEMENT

Bacteriophages, viruses that often kill their bacterial host during replication, are being developed as phage-based therapies to treat drug-resistant bacterial infections in humans and in agriculture (1, 2).

**Fig 1:**
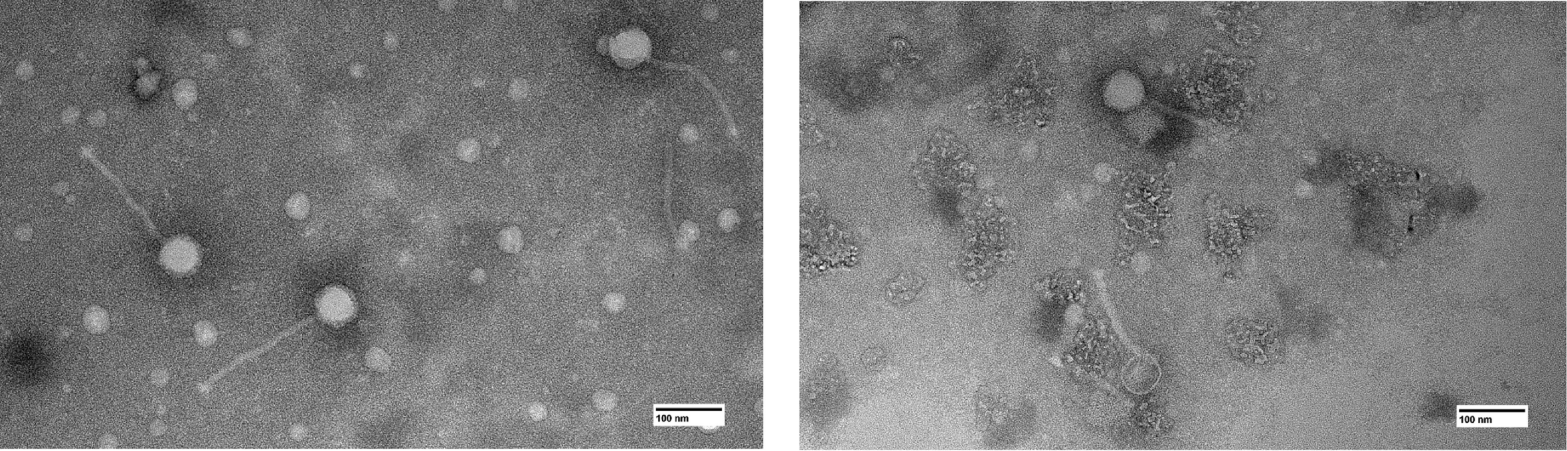
Shambre1 on the left, Renna12 on the right. Negative-staining (1% uranyl acetate) transmission electron microscopy revealed Shambre1 and Renna12 to be siphoviruses. Shambre1 has a tail length of 190–210 nm and an isometric head diameter of 50–55 nm (*n* = 6), while Renna12 has a tail length of 150–200 nm and an isometric head diameter of 50–55 nm (*n* = 6). The scale bar is 100 nm.

We report the discovery and characteristics of two phages, Shambre1 and Renna12, both isolated from soils in Bismarck, ND; Shambre1 was isolated 3 inches deep under a tree (46.780869 N, 100.780869 W) while Renna12 was isolated near a fence (46.730379 N, 100.752325 W) using *Arthrobacter globiformis* B-2979-SEA and standard procedures (3). Briefly, each soil sample was suspended in peptone-yeast-calcium liquid broth, inoculated with *A. globiformis*, and grown at 30°C with shaking. After 3 days of incubation, the culture was filtered (0.2 mm pore size) and plated in top agar with *A. globiformis*. After 48–72 hours at 30°C, Shambre1 formed clear plaques with a cloudy perimeter 1 mm in diameter, whereas Renna12 formed clear plaques 3 mm in diameter. Negative staining transmission electron microscopy revealed both phages to have siphovirus morphologies (Fig. 1).

Double-stranded DNA was isolated from Shambre1 and Renna12 using the Promega Wizard DNA Cleanup kit and sequenced using an Illumina MiSeq Sequencer (v3 reagents). The library was prepared using the NEB Ultra II Library Kit, yielding 279,545 single-end 150 bp reads for Shambre1 (122-fold coverage) and 24,8416 single-end 150 bp reads for Renna12 (481-fold coverage). Raw reads were assembled and checked for completeness using Newbler v2.9 and Consed v29, using default parameters (4). The resulting genome for Shambre1 was 42,211 bp long, with 3′ 10 base single-stranded ends (CGCCGGGGTA) and a G + C content of 65.4%. Renna12 had a genome of 39,179 bp with 3′ 12 base single-stranded ends (GAGTTGCCGGCA) and a G + C content of 66.0%. Based on gene content similarity of at least 35% to phages in the Actinobacteriophage database (https://phagesdb.org/), Renna12 was assigned to the cluster AS (5, 6). Shambre1 does not meet this threshold with any phage in the database, therefore classified as a singleton.

Genome annotation was performed with DNA Master (cobamide2.bio.pitt.edu) and PECAAN (7), both of which utilize Glimmer-v3.02 (8) and Genemark-v3.25 (9) to predict potential open-reading frames, as well as Phamerator (10) and Starterator (http://phages.wustl.edu/starterator/). ARAGORN (11) and tRNAscan-SE (12) were used to detect the presence of tRNAs. Gene functions were predicted with BLASTp (13) searches against the Actinobacteriophage and NCBI non-redundant databases as well as HHPred (v3.18) searches against the PDB mmCIF70, Pfam-A, and NCBI Conserved Domain databases (14).

Annotation revealed the genome sequence of Shambre1 to contain 67 candidate protein-coding genes, 28 of which could be assigned putative functions. Most genes encoding structure and assembly functions were located on the left half of the genome, while those involved in DNA metabolism and lysis are on the right half of the genome. No immunity repressor or integrase functions could be identified. With the exception of two clusters of genes (*28–36, 57–60*), all genes are transcribed rightwards. Renna12 contains 1 tRNA and 71 candidate protein-coding genes, of which 39 genes could be assigned putative functions, including an immunity repressor and tyrosine integrase shared with other cluster AS phages. All Renna12 genes are transcribed rightwards, except for genes *25–36*.

## Data Availability

Renna12 is available at GenBank accession number OP297552 and Sequence Read Archive (SRA) no. SRX14989443. Shambre1 is available at GenBank accession number OP297545 and Sequence Read Archive (SRA) no. SRX14485089.
